# Supplementary material for: Inactivation of SARS-CoV-2 through Treatment with the Mouth Rinsing Solutions ViruProX® and BacterX® Pro
Source: Microorganisms. 2021 Mar 3;9(3):521. doi: 10.3390/microorganisms9030521 (PMC8002120; doi:10.3390/microorganisms9030521)
Supplement: Supplementary file 1 [file microorganisms-09-00521-s001.pdf]

Supplementary material

Table S1

Test solution	Composition	Manufacturer	Lot	Recommendation of the manufacturer
ViruProX®	Aqua, Propylene Glycol, Glycerin, PEG-40, Hydrogenated Castor Oil, Aroma, Hydrogen Peroxide (1.5%), Cetylpyridinium Chloride (0.05%), Sucralose, Erythritol, Sodium Saccharin	Dr. Wittmann GmbH & Co KG	Lot.: 2005191	gargle and rinse with at least 10mL undiluted solution for 40 s
BacterX® pro	Aqua, Glycerol, Propylene Glycol, PEG-40, Hydrogenated Castor Oil, Sucralose, Chlorhexidine Digluconate (0.1%), Cetylpyridinium Chloride (0.05%), Xylite, Aroma, Sodium Fluoride (0.005%), CI 42051	Dr. Wittmann GmbH & Co KG	Lot.: 2006221	rinse the mouth with undiluted solution for 20-30s
0.05% CPC		Dr. Wittmann GmbH & Co KG	Lot: K93839740945<br><br>Preparation Date: 14.07.2020	
0.1% CHX		Dr. Wittmann GmbH & Co KG	Lot.: 3-8962-9-02-17,<br><br>Preparation Date: 14.07.2020	
1.5% H_2O_2		Dr. Wittmann GmbH & Co KG	1200203404, Preparation Date: 14.07.2020	
